# Development and Validation of LC-MS/MS Method for Determination of Cytisine in Human Serum and Saliva

**DOI:** 10.3390/ijms242015364

**Published:** 2023-10-19

**Authors:** Karol Wróblewski, Małgorzata Szultka-Młyńska, Ryan J. Courtney, Bogusław Buszewski, Piotr Tutka

**Affiliations:** 1 Laboratory of Commercial and Non-Commercial Clinical Trials, University of Rzeszów, Kopisto 2a, 35-959 Rzeszow, Poland; kwroblewski@ur.edu.pl; 2 Laboratory for Innovative Research in Pharmacology, University of Rzeszów, Kopisto 2a, 35-959 Rzeszow, Poland; 3 Interdisciplinary Center for Preclinical and Clinical Research, University of Rzeszów, Werynia 2A, 36-100 Kolbuszowa, Poland; 4 Department of Environmental Chemistry and Bioanalytics, Faculty of Chemistry, Nicolaus Copernicus University, Gagarina 7, 87-100 Torun, Poland; szultka.malgorzata@wp.eu; 5 National Drug and Alcohol Research Centre, University of New South Wales, Sydney, NSW 2052, Australia; r.courtney@unsw.edu.au; 6 Prof. Jan Czochralski Kuyavian-Pomeranian Science and Technology Center, Parkowa 1, 87-134 Przysiek, Poland; bbusz@chem.umk.pl; 7 Department of Experimental and Clinical Pharmacology, University of Rzeszów, Kopisto 2a, 35-959 Rzeszów, Poland

**Keywords:** cytisine, serum, saliva, LC-ESI-QTOF-MS, nicotine addiction

## Abstract

Cytisine (CYT) is a quinolizidine alkaloid used for nicotine addiction treatment. Recent clinical trial data regarding cytisine confirm its high effectiveness and safety as a smoking cessation treatment. CYT’s popularity is growing due to its increased availability and licensing in more countries worldwide. This increased use by smokers has also resulted in an urgent need for continued drug research, including developing appropriate analytical methods for analyzing the drug in biological samples. In this study, a simple, fast, and reliable method combining hydrophilic interaction liquid chromatography and electrospray ionization quadrupole time of flight mass spectrometry (HILIC/ESI-QTOF-MS) for the determination of CYT in human serum and saliva was developed and validated. This was undertaken after the previous pre-treatment of the sample using solid-phase extraction (SPE). A hydrophilic interaction liquid chromatography (HILIC) column with a silica stationary phase was used for chromatographic analysis. In a linear gradient, the mobile phase consisted of acetonitrile (ACN) and formate buffer at pH 4.0. The proposed method was fully validated and demonstrated its sensitivity, selectivity, precision, and accuracy. The method was successfully applied to determine CYT in serum and, for the first time, in saliva. The findings indicate that saliva could be a promising non-invasive alternative to measure the free concentration of CYT.

## 1. Introduction

Cytisine (CYT) is a quinolizidine alkaloid found in plants of the Leguminosae (Fabaceae) family: for example, in *Laburnum anagyroides* (Golden Rain). The chemical structure of CYT is similar to nicotine, and the drug is marketed in tablet and capsule form. It is predominately used for nicotine addiction treatment in Europe, Canada, and Central Asia [1,2]. CYT acts as a partial agonist at nicotinic acetylcholine receptors (nAChRs), which are believed to mediate the effects of nicotine [3]. In recent years, clinical trials have found CYT to be superior to placebo [4,5,6] and nicotine replacement therapy [7], while recent findings for CYT compared to varenicline, which is considered to be the most effective drug for smoking cessation that is currently available, are mixed [2,8,9]. In addition, CYT causes fewer adverse events than varenicline [9,10]. With more high-quality trials that meet evidence-based medicine practice standards, scientists’ and physicians’ interest in CYT as an effective, safe, and accessible smoking cessation treatment has grown [2]. CYT could also be developed as a potential therapeutic candidate for ethanol consumption and human dependence [10,11,12].

CYT at standard dosing for smoking cessation is administered for 25 days, starting with a dose of 1.5 mg every 2 h for the first 3 days (9 mg/day), then gradually tapering to two doses per day on days 21–25 (3 mg/day). A treatment schedule longer than 25 days with cytisine may also be of benefit to reduce post-treatment smoking relapses [9,13,14], but extended dosing has not yet been licensed for use in any country.

There are limited data available on studies of CYT levels in human body fluids and only a few pharmacokinetic studies. A single-dose pharmacokinetic study was conducted after 1.5, 3, and 4.5 mg [15,16]. Studies have shown that CYT is not metabolized (or its metabolism is negligible) and renally eliminated. The average elimination half-life of CYT is 4.8 h [15]. There is one published work related to measuring plasma CYT concentrations in patients instructed to follow the 25-day standard dosing regimen [17].

To date, few methods have been developed for the determination of CYT in human serum/plasma [15,18,19], whole blood [20], urine [15,18,21], and material collected posthumously (blood, stomach contents, bile, liver, and brain) [22]. Most of the above methods were developed for the detection and/or determination of CYT in humans after poisoning with plant material or tablet overdose [18,19,20,21,22]. The quantitation of CYT in human biological samples has been performed using high-performance liquid chromatography coupled with mass spectrometry (HPLC-MS) or tandem mass spectrometry (HPLC-MS/MS) [15,16,17,18,19,20,22]. The pre-treatment of the sample before chromatographic analysis was performed by solid-phase extraction (SPE) [15,16,17,18,23], liquid–liquid extraction (LLE) [21,22], or protein precipitation [15].

The retention mechanism of CYT in different chromatographic conditions has been studied in our earlier studies [23]. The evaluation of various chromatographic systems for the analysis of CYT in serum, saliva, pharmaceutical formulation [23], and plant extracts [24] by HPLC with various detection techniques has also been performed. The findings can be applied for the development of analytical methods for the quantitative and qualitative analysis of CYT in various biological samples.

The first detection of CYT in saliva was conducted by Wróblewski et al. [23]. The developed method allows for the qualitative analysis of CYT by HPLC-DAD (in high concentration) and LC-QqQ-MS within a therapeutic concentration range. Different analytical techniques for the detection of CYT in various matrices have also been compared. The findings indicated that LC-MS/MS was the most suitable for CYT analysis in biological samples due to the sensitivity of the assays.

There are still gaps in the knowledge about CYT in various areas of preclinical and clinical research. Further studies are necessary, including those related to the development of reliable analytical methods for CYT determination in various biological samples, drug fate studies in the body, and pharmacokinetic studies in various patient groups.

A method for determining drug concentration in saliva is a viable and complementary alternative for measurements in blood or urine. The undeniable advantage of this method is its non-invasive nature and safe sampling. Moreover, the concentration of free drugs in saliva may better correlate with their therapeutic effect than their total plasma concentrations [25]. There is currently no established method to measure CYT concentration in the saliva.

This study aimed to develop a high-performance liquid chromatography–tandem mass spectrometry method (HPLC-MS/MS) for the qualitative and quantitative analysis of CYT in serum and, for the first time, in saliva. The proposed method was successfully applied to determine CYT in human body fluids obtained from study participants treated with CYT in a standard dosing regimen.

## 2. Results and Discussion

### 2.1. HILIC/ESI-QTOF-MS/MS Method Development

Chromatographic analysis was performed using a HILIC column with a silica stationary phase. The optimization chromatographic systems for the analysis of CYT were performed in our earlier research [23]. CYT is a very polar primary compound with weak retention in reverse-phase chromatographic systems. For this reason, it was necessary to apply mobile phases containing a small amount of organic modifier in water or only water with appropriate additives to reduce peak tailing, such as diethylamine, sodium salts, ionic liquids, or formic acid. Many of these systems are unsuitable for quantifying CYT by LC-MS. A good alternative is HILIC, which offers essential advantages for the mass spectrometric detection of small polar compounds compared to reversed-phase chromatography. HILIC is suitable for the LC-MS analysis of polar drugs or their metabolites [26,27]. The higher organic content of the eluent in HILIC supports the efficient evaporation of the solvent, enhancing sensitivity and altering ion suppression. According to our previous study [23], HILIC systems can be helpful for the chromatographic analysis of CYT, and the best result was obtained on the HILIC-A column (silica stationary phase) with the mobile phase containing ACN and formate buffer at pH 4.0. Based on the above results, a new chromatographic system was developed that is suitable for determining CYT in both serum and saliva samples by HPLC-QTOF-MS.

**Figure 1:**
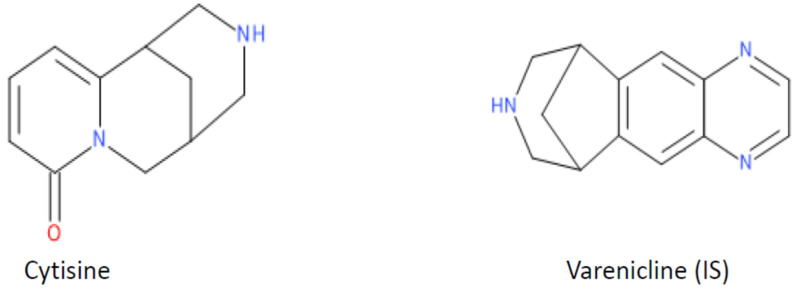
Chemical structure of CYT and varenicline (IS).

Thus, a HILIC-A column was used as the stationary phase, and the mobile phase contains ACN (eluent A) and a 5 mM formate buffer at pH 4.0 (eluent B) in linear gradient elution mode. Varenicline was selected as the internal standard (IS). It has a chemical structure similar to the CYT (Figure 1) and is not expected to be naturally present in analyzed samples. The protonated mass [M+H]^+^ of varenicline was 212.1176. The retention time (t_R_) for CYT was 3.64 min, and for IS was 2.89 min. The total time required for a single analysis was 8.0 min. The proposed method allows us to obtain a good resolution, symmetric peak shapes, good analyte signal response, and short run times. This is the first method for the determination of CYT in biological samples using HILIC. To date, as reported in the literature [15,16,17,18,19,20,22], the determination of CYT in human body fluids has been performed using an RP18 stationary phase, which gives poor retention due to the polar character of CYT.

For sample pre-treatment, prior to LC-QTOF-MS analysis, a SPE procedure developed in our earlier work [23] was adapted and subjected to a proper validation procedure with an appropriate number of experimental repetitions. The obtained recoveries are higher than those presented in the literature for the validated analytical method quantification of CYT in human biological samples [15,16,17,20,22].

**Figure 2:**
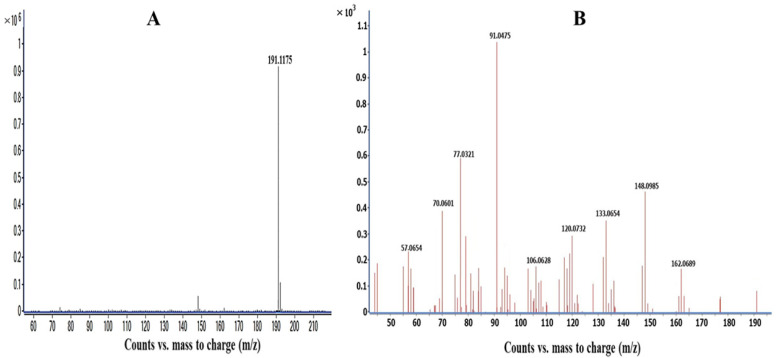
The MS (**A**) and MS/MS (**B**) spectra obtained for CYT.

The ESI source conditions were appropriately optimized to obtain a good signal-to-noise (S/N) ratio and high sensitivity. In order to maximize the ionization in the source, the flow and temperature of the drying gas, the nebulizer gas flow, capillary, ionization, and skimmer were optimized. The collision energy (CID) was optimized corresponding to the CYT fragmentation. A range of collision energies from 10 to 40 eV was tested. The obtained results show noticeable differences between the MS spectra depending on the CID energy applied. A sufficient CYT fragmentation was obtained for 25 eV. The [M+H]^+^ ions of CYT are presented in Table 1. The MS and MS/MS spectra of cytisine are shown in Figure 2.

The developed method also enables the detection of cotinine in serum and saliva. Cotinine is the major metabolite of nicotine, has a much longer half-life (16 h), and is present in body fluids at much higher levels than nicotine [28]. For these reasons, cotinine is one of the main biochemical markers used for measuring exposure to cigarette smoke and has been widely applied for assessing smoking status [29]. The protonated mass [M+H]^+^ was 177.1018 and the characteristic fragment ions obtained according to the MS/MS fragmentation pattern (collision energy, 25 eV) included the following masses: 149.0604, 146.0508, 120.0694, 118.0560, 98.0529, 80.0423, 70.0590, and 53.0336. These, however, are only preliminary studies to determine the suitability of the developed SPE procedure and LC-MS/MS method for detecting cotinine as an added value and benefit during CYT quantification. Additional validation studies are needed to determine the method’s detection limit, accuracy, and precision.

In recent years, an increase in the role of liquid chromatography coupled with high-resolution mass spectrometry in quantitative analysis (LC-HRMS) has been observed. Therefore, there are an increasing number of quantitative methods using LC-Q-TOF-MS. The precision of Q-TOF instruments has been shown to be as good as that of triple quadrupole [30]. The major advantage of performing targeted analyses using high-resolution mass spectrometry (HRMS) is the ability to simultaneously acquire untargeted data that can be mined retrospectively for additional compounds of interest not originally considered in targeted method development. As the latest research results show, Q-TOF technology can be successfully used for robust and accurate quantification with adequate sensitivity [31,32,33]. The results of our study confirm the usefulness of LC-Q-TOF-MS in quantification and represent a new perspective of the utility of LC-HRMS systems in the future.

### 2.2. Method Validation

The proposed method was validated according to the recommendations of to the European Medicines Agency (EMA) [34], International Council for Harmonization of Technical Requirements for Pharmaceuticals for Human Use (ICH) [35], and the Food and Drug Administration (FDA) Bioanalytical Method Validation Guidance for Industry [36] guidelines.

#### 2.2.1. Selectivity

**Figure 3:**
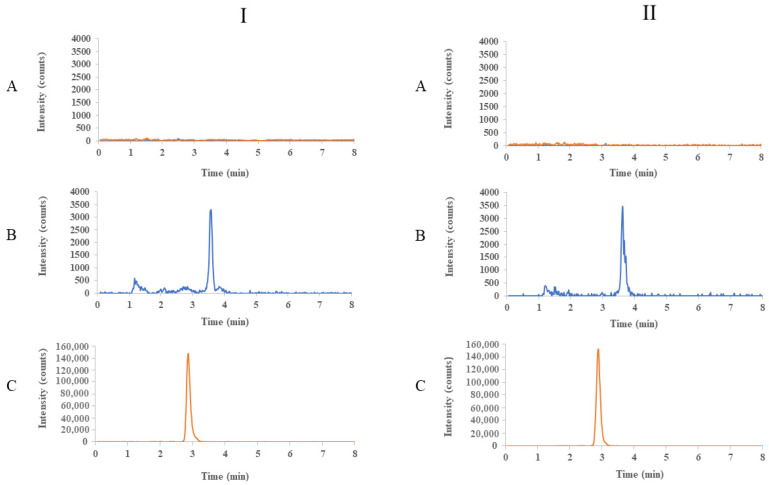
Chromatograms (EIC) obtained for serum (**I**) and saliva (**II**): (**A**) blank, (**B**) spiked with CYT at LLOQ level (1 ng/mL), and (**C**) spiked with IS (50 ng/mL).

The evaluation of selectivity demonstrated no significant interference for the CYT and IS from matrix components in the serum and saliva samples. The responses of any interfering components at the retention times of the CYT were not greater than 20% of the analyte response at the LLOQ. Figure 3 shows chromatograms obtained for human serum and saliva: blank, spiked with CYT at LLOQ level (1 ng/mL), and spiked with IS (50 ng/mL).

#### 2.2.2. Linearity

To evaluate linearity, calibration curves with six concentration points were prepared. The calibration curves were constructed by analyzing samples previously treated with the developed SPE procedure. The developed LC–MS/MS method was linear within the tested calibration range of 1–100 ng/mL (r = 0.9999) for saliva and 1–200 ng/mL (r = 0.9999) for serum. Equations for the calibration curves and regression coefficients are presented in Table 2.

#### 2.2.3. Lower Limit of Detection (LLOD) and Lower Limit of Quantification (LLOQ)

The obtained LLOD was 0.5 ng/mL for serum and 0.35 ng/mL for saliva samples. The LLOQ was 1 ng/mL for both serum and saliva samples (Table 2).

#### 2.2.4. Accuracy and Precision

The intra- and inter-assay validation data are summarized in Table 3. The values of the intra-day accuracy were obtained in the range of 93.79–99.39% and 95.93–108.95% for serum and saliva samples, respectively. The inter-day accuracy value was in the range of 94.63–107.05% and 98.21–102.46% for serum and saliva samples, respectively. Intra-day precision expressed as % CV ranged from 4.73 to 13.82% for serum and from 5.82 to 13.01% for saliva samples. Inter-day precision expressed as % CV ranged from 2.60 to 15.17% and from 2.62 to 14.34% for serum and saliva samples, respectively. The results show acceptable criteria: 85–115% (80–120% for LLOQ) for accuracy and ≤15% (≤20% for LLOQ) for precision.

#### 2.2.5. Recovery and Matrix Effect

The obtained recoveries were in the range of 92.95–95.61% for serum samples and 96.30–98.07% for saliva samples (Table 4). The use of the developed SPE procedure provides higher recoveries compared to other methods of CYT isolation described in the literature for human body fluids [15,20,21,22]. This was also stated in our earlier work [23]. The matrix effect ranged from 100.35 to 110.63% for serum samples and from 103.67 to 113.95% for saliva samples (Table 4).

#### 2.2.6. Stability

##### Stability of Stock Solution

The obtained recovery (n = 6) was higher than 99% in all cases. No significant variation in the recovery was observed and no degradation product was detected. Therefore, the methanol solution of CYT is stable in the presented conditions (Table 5).

##### Stability of Biological Samples

The results of the stability tests in biological samples are presented in Table 6. The results are well within the acceptable limits.

### 2.3. Application of HILIC/ESI-QTOF-MS Method for Determination of CYT in Serum and Saliva

**Figure 4:**
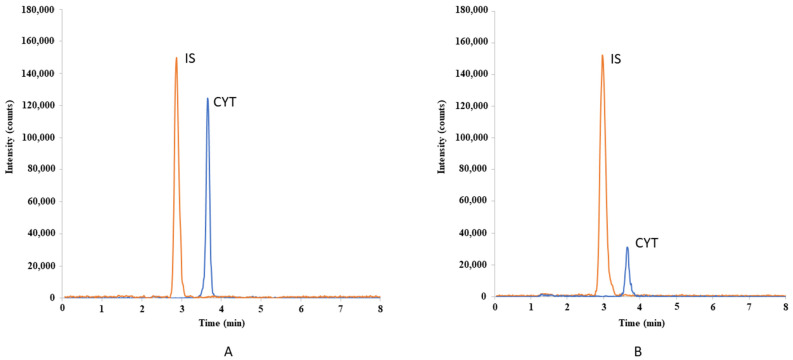
Chromatograms obtained for CYT determined in serum ((**A**); 37.15 ng/mL) and saliva ((**B**); 10.32 ng/mL) sample collected 10 h after the first dose of drug.

The developed method has been successfully applied to the determination of CYT in serum and saliva obtained from patients using CYT according to the standard dosage regimen. The determination of CYT in saliva was performed for the first time. The developed method was also applied for the first time for the determination of CYT in serum and saliva samples obtained from a 60-year-old man who used electronic nicotine delivery system (ENDS) and was treated with a standard dosage regimen of CYT. The participant expressed a willingness to stop vaping. Patient characteristics are shown in Table S1. The measured concentration of carbon monoxide (CO) in exhaled air using a smokerlyzer was 3 ppm on the day of the study. This confirmed that the patient was not smoking traditional cigarettes as per the threshold adopted. Using the developed analytical method, cotinine was also detected in this participant, which confirms nicotine use. On day 1 of the study, blood and saliva samples were collected for the determination of CYT concentration at 30 min and 1, 2, 4, 6, 8 and 10 h after the first dose of CYT. Figure 4 shows examples of chromatograms obtained for CYT determined in serum and saliva samples from this patient.

**Figure 5:**
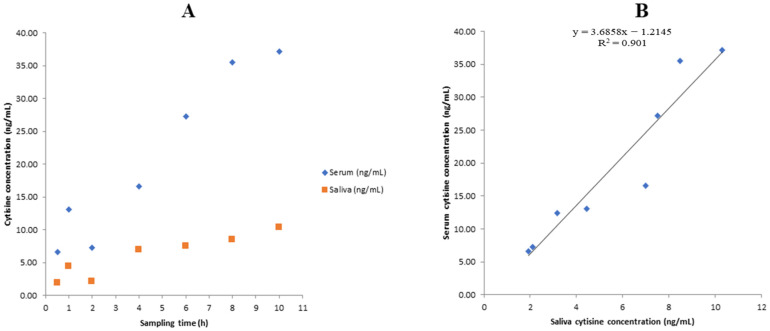
(**A**) The concentrations of CYT in serum and saliva (samples collected on the first day of the treatment). (**B**) The correlation between serum and saliva CYT concentration. The CYT concentrations were measured in samples obtained from an ENDS user treated with CYT at the standard dosing regimen.

On day 1, the CYT concentrations in the biological fluids of the ENDS user increased with repeated dosing (1.5 mg every 2 h) (Table 7, Figure 5A), and accumulation of the drug was observed. Measurement of CYT concentration started on samples collected 0.5 h after the first dose of the drug (1.5 mg). The concentration of the drug was 6.63 in serum and 1.93 ng/mL in saliva. The serum and saliva concentrations measured before the last dose of the day (at 10 h after the first dose) were 37.15 and 10.32 ng/mL, respectively. The value of the serum concentration of CYT measured 10 h after the first dose of the day was lower than the value reported in plasma by Jeong et al. (mean ± SEM: 50.8 ± 4.7 ng/mL, participants: traditional cigarette smokers) [17]. On day 4 (14 h after the last dose), the serum and saliva CYT concentrations were 12.44 and 3.17 ng/mL, respectively. The ratio of the saliva concentration to the serum concentration of CYT was 0.3 ± 0.06 (%CV = 19.34). As shown in Figure 5B, a linear relationship was observed between the saliva and serum concentration of CYT (R^2^ = 0.901). A good correlation, n, between the saliva and serum levels of CYT may allow for predicting serum CYT concentration from saliva measurements with high accuracy. However, this was only a case study, and research with an appropriate population size needs to be carried out in the future. The patient did not attend the last scheduled appointment after the end of therapy (day 26). Thus, it was not possible to confirm the patient’s abstinence and the effectiveness of the applied treatment.

The correlation of serum and salivary cytisine concentrations and the evaluation of the safety of cytisine in smokers and non-smokers will be described in another work.

## 3. Materials and Method

### 3.1. Chemicals and Reagents

Desmoxan (capsules, CYT 1.5 mg) and standards of CYT were purchased from Aflofarm (Pabianice, Poland). Hydrochloric acid, ammonium 25%, formic acid (98–100%), ammonium formate, ACN, methanol (MeOH), and water for LC-MS were obtained from Merck (Darmstadt, Germany). Standards of cotinine and varenicline tartrate (internal standard, IS) were purchased from Sigma-Aldrich (Saint Louis, MO, USA).

### 3.2. LC-ESI-Q-TOF-MS Conditions

The quantification of CYT and the detection of cotinine were carried out with the use of the UHPLC Agilent 1290 Series system (Agilent Technologies, Waldbronn, Germany) equipped with a 6540 UHD accurate mass Q-TOF detector, an ESI interface, and Mass Hunter software for instrumental control and data collection. The manufacturer’s calibration solution was used for the calibration of the mass spectrometer before analysis. Chromatographic HILIC A column (3 mm × 100 mm, 3 µm, Agilent Technologies, Waldbronn, Germany) was maintained at 25 ± 0.5 °C. The mobile phase was composed of ACN (mobile phase A) and 5 mM of formate buffer at pH 4.0 (mobile phase B). A linear gradient elution from 70% to 60% mobile phase A in 8 min with a flow rate of 0.4 mL/min was applied. The injected sample volume was 10 µL. The retention time for CYT was 3.64 min and for IS 2.89 min. The electrospray ion source operating in the positive ion mode (ESI(+)) was applied in quadrupole time-of-flight mass spectrometric analyses. The following set of operation parameters was used: the drying gas temperature (DGT), 260 °C; the shielding gas temperature (SGT), 305 °C; the fragmentor voltage (FV), 175 V; the capillary voltage (CV), 3.5 kV; the octopole voltage (OV), 750 V; and the skimmer voltage (SV), 45 V. Nitrogen was applied as nebulizing (35 psig), drying (6 L/min), collision, and curtain gas. The used collision energy was 25 eV. High-purity nitrogen gas was applied for the nebulizer/DuosprayTM (Agilent Technologies, Waldbronn, Germany). The Q-TOF and information-dependent acquisition scan was performed with a mass range of 40 to 400 *m*/*z*. The MassHunter Workstation software (B.04.01, Agilent Technologies, Waldbronn, Germany) was used for the data acquisition and processing. The data were also further processed using Microsoft Excel.

### 3.3. Serum and Saliva Sample Collection

Serum and saliva samples were collected from smokers, non-smokers, and ENDS users who were treated with CYT at a dose of 1.5 mg according to the standard dosing regimen: starting with 1 dose (Desmoxan, caps. 1.5 mg) every 2 h for the first 3 days (9 mg/day), then gradually tapering to two doses per day on days 21–25 (3 mg/day). This was a single-center study conducted at MEDYK’ Medical Center in Rzeszow, Poland. The study protocol was approved by the Bioethical Committee of the Medical University of Lublin (approval number KE-0254/165/2018). Participants signed informed consent.

Twelve non-smokers (aged between 22 and 53 years, eight women and four men), eleven smokers (aged between 25 and 61 years, six women and five men), and one ENDS user (a 60-year-old man), motivated to quit smoking or vaping, respectively, took part in the study. Participants were excluded from the study if they were pregnant or breastfeeding or had no severe comorbidities, including liver and kidney diseases. Cigarette smokers were included if they had smoked at least 10 cigarettes per day over the past 12 months with a carbon monoxide concentration in exhaled breath of at least 10 ppm confirmed at screening.

Study participants were asked not to eat food or brush their teeth two hours before the test and to drink beverages for 20 min before saliva collection. Participants were instructed to not drink caffeinated beverages (energizing drinks, coffee) for 12 h before the test. Participants were asked to rinse their mouths three times with a small amount of deionized water 10 min before saliva collection. Unstimulated, whole saliva (at a volume of about 1–2 mL) was collected by the spitting method. Participants spat saliva into a plastic container in no more than 5 min. Then, the saliva was frozen at −80 °C. Saliva was transported under non-thawing conditions.

**Figure 6:**
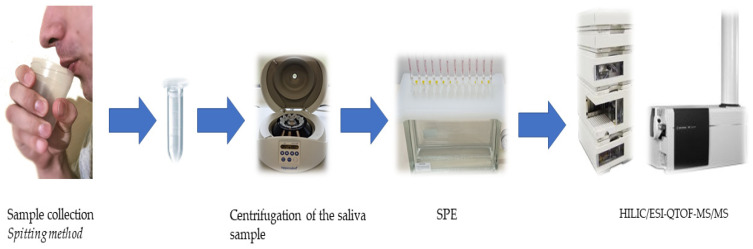
A schematic of saliva collection and sample preparation prior to HPLC analysis.

The scheme of the procedure for saliva collection, pre-treatment, and sample preparation for chromatographic analysis is shown in Figure 6.

### 3.4. Preparation of the Serum Samples

The collected blood (5 mL) was incubated at room temperature (15–24 °C) until a clot formed (approximately 30–40 min). Then, the blood was centrifuged in a centrifuge in accordance with the parameters recommended by the manufacturer of blood collection systems (for 15 min at 2000 rpm). The serum was transferred to a sterile plastic tube with an airtight stopper. The serum tube was frozen at −80 °C. Serum was transported under non-thawing conditions.

### 3.5. Extraction Procedure

Solid-phase extraction (SPE) was applied for the isolation of investigated compounds from serum and saliva samples. SPE was performed using Strata X-C cartridges (30 mg/mL, Phenomenex) and an SPE chamber—Baker SPE—12G (J.T. Baker, Philipsburg, PA, USA). The extraction procedure was adopted from the method developed by Wróblewski et al. [23]. The sample (0.25 mL) was diluted with 0.25 mL of 0.1 M HCl. Extraction columns were activated and conditioned with 0.5 mL of MeOH and next 0.5 mL of 0.1M HCl. Then, the sample was introduced to the column at a speed of 1 mL/min. The column was then prewashed with 0.5 mL of 0.1 M HCl followed by 0.5 mL of MeOH, and dried by applying a vacuum for 5 min. The investigated compounds were eluted twice with 0.5 mL of eluent containing MeOH and 25% ammonium solution in a ratio of 95:5. The sample was evaporated to dryness and dissolved in 50 μL of MeOH. A total of 10 μL of the eluate was injected into the HPLC column.

### 3.6. Preparation of Stock Solution and Working Solutions

The stock standard solutions of CYT and IS were prepared in MeOH at a concentration of 0.2 mg/mL by dissolving 10 mg of the drug or IS in 50 mL of MeOH. The solutions were stored at −20 °C and protected from light. The working standard solutions were prepared by diluting stock solutions in MeOH before analysis.

### 3.7. Method Validation

The validation study was performed using spiked human serum and saliva according to the recommendations of the EMA [34], ICH [35], and FDA [36] guidelines and included the evaluation of linearity, lower limit of detection (LLOD), lower limit of quantification (LLOQ), selectivity, extraction recovery, matrix effect, precision, and accuracy.

#### 3.7.1. Linearity

Linearity was evaluated by preparing calibration curves plotting the peak area ratios of CYT to IS against the concentrations of CYT. Blank serum or saliva samples were spiked with suitable amounts of CYT standard. Samples were pre-treated by SPE and determined by the HPLC-MS method described above. Calibration curves were constructed by analyzing spiked samples at seven concentrations, ranging from 1 to 200 ng/mL and 1 to 100 ng/mL for serum and saliva, respectively. The calibration curve included a zero sample (spiked with IS), a blank sample (no IS), and non-zero samples including the LLOQ. The standard curve was prepared on each day of the analyses.

#### 3.7.2. Selectivity

Selectivity was assessed by analyzing the saliva and serum samples from six different participants (smokers and non-smokers) to investigate the potential interferences with the signals of CYT and IS. The extent of interferences originated by endogenous sample components at the specific retention time of each analyte was evaluated by comparing chromatograms of various batches of human serum and saliva with and without spiking CYT and IS.

#### 3.7.3. Lower Limit of Detection (LLOD) and Lower Limit of Quantification (LLOQ)

Limit of detection (LOD) was calculated by the determination of the signal-to-noise ratio of 3:1. The LLOQ was evaluated based on a signal-to-noise ratio of at least 5:1 with acceptance, precision, and accuracy within 20% of the nominal values. The LLOQ was used as the lowest calibration standard.

#### 3.7.4. Precision and Accuracy

Intra-day precision and accuracy were calculated on the same day by analyzing six replicates of quality control (QC) samples at four concentration levels, low QC (LLOQ), two mid-QCs, and high QC: 1 ng/mL, 20 ng/mL, 90 ng/mL, and 180 ng/mL for serum samples and 1 ng/mL, 10 ng/mL, 40 ng/mL, and 90 ng/mL for saliva samples. The inter-day accuracy and precision were determined on three separate days by analyzing QC samples at each concentration level.

#### 3.7.5. Extraction Recovery and Matrix effects

Extraction recovery and matrix effects were evaluated at four concentration levels (low QC, two mid-QCs, and high QC) according to the following formulas:

Matrix effect (%) = B/A × 100

Recovery (%) = C/B × 100

A = external solution peak area, B = post-extraction sample peak area, and C = extracted matrix peak area.

### 3.8. Drug Stability

#### 3.8.1. Stability of Stock Solution

The stability of the stock solution of CYT in MeOH (0.2 mg/mL) was evaluated after 1 year at a temperature of −20 °C, after two weeks at a temperature of 4 °C, and 24 h at room temperature. The solution was stored in glass containers protected from light.

#### 3.8.2. Stability of Biological Samples

Drug stability in a biological fluid was evaluated by analyzing human serum and saliva samples spiked with CYT that were exposed to different conditions (temperature and time). Tests were conducted at three concentration levels (1, 10, and 90 ng/mL for saliva samples and 1, 20, 180 ng/mL for serum samples). These results were compared with those obtained from freshly prepared saliva and serum samples. Freeze–thaw stability was evaluated after three complete free/thaw cycles (−20 to 25 °C). Short-term temperature stability was assessed by analyzing samples kept at ambient temperature (25 °C) for 24 h as well as at −4 °C for 48 h. Long-term stability was assessed using serum and saliva samples stored at −20 °C for 30 days. To evaluate the post-preparative stability, biological samples were extracted and kept in the autosampler at ambient temperature (25 °C) for 8 h before the injection.

## 4. Conclusions

A simple and fast HILIC/ESI-QTOF-MS/MS method has been developed and validated to determine CYT in human serum and, for the first time, in human saliva. The results indicate that the method is accurate, precise, and sensitive. A simple SPE procedure obtained good recoveries. The accepted method was successfully applied in the quantitative analysis of CYT in human serum and saliva. A good correlation between the saliva and serum levels of CYT was reported in a case study of an ENDS user treated with CYT. This was the first documented use of CYT in the treatment of nicotine addiction caused by an electronic nicotine delivery system. Our results indicate that saliva can be used as an alternative matrix to blood to monitor CYT concentration. However, more research is needed on the appropriate size of the population group. This will be the topic of our subsequent work in which the described method will be applied.

## Figures and Tables

**Table 1:** MS parameters for cytisine.

Compound	Chemical Formula	Molecular Ion [M+H]^+^	Fragment Ions	Collision Energy (eV)
Cytisine	C_11_H_14_N_2_O	191.1175	162.0689, 148.0985, 133.0654, 120.0732, 119.0580, 106.0628, 91.0475, 77.0321, 57.0654	25

**Table 2:** Parameters of calibration curves for quantitative analysis of CYT in serum and saliva: calibration curves’ equations, concentration range, regression coefficients (r), the lower limit of detection (LLOD), and the lower limit of quantitation (LLOQ).

Sample Type	Regression Equation	Range	r	LLOD (ng/mL)	LLOQ (ng/mL)
Saliva	y = 0.0092x + 0.0028	1–100 ng/mL	0.9999	0.35	1
Serum	y = 0.0238x + 0.0042	1–200 ng/mL	0.9999	0.5	1

**Table 3:** Validation data: intra-day and inter-day accuracy and precision.

Type of Sample	Concentration Added (ng/mL)	Intra-Day			Inter-Day		
		Concentration Found (ng/mL)	Accuracy (%)	Precision (%CV)	Concentration Found (ng/mL)	Accuracy (%)	Precision (%CV)
Serum	1	0.94	93.79	13.82	0.95	94.63	15.17
	20	19.24	96.19	7.51	21.41	107.05	9.59
	90	89.45	99.39	6.12	87.35	97.05	6.10
	180	176.41	98.01	4.73	184.55	102.53	2.60
Saliva	1	1.09	108.95	13.01	1.02	102.26	14.34
	10	9.92	99.17	6.81	10.25	102.46	7.56
	40	38.37	95.93	7.84	39.29	98.21	8.02
	90	87.75	97.50	5.82	90.42	100.47	2.62

**Table 4:** Recovery and matrix effect obtained for CYT in serum and saliva samples.

Type of Sample	Concentration Added (ng/mL)	Recovery (%)	SD *	Matrix Effect (%)	SD *
Serum	1	95.38	8.65	110.63	8.05
	20	92.95	5.63	108.50	8.70
	90	93.21	5.73	103.88	5.30
	180	95.61	4.43	100.35	3.67
Saliva	1	96.30	7.73	113.95	6.13
	10	96.79	3.73	107.35	5.23
	40	97.74	4.03	107.40	5.84
	90	98.07	4.49	103.67	4.42

* SD—standard deviation.

**Table 5:** Stability of CYT in the stock solution (n = 6).

Conditions	Time	Recovery	SD *
−20 °C	1 year	99.99%	1.01
4 °C	2 week	99.81%	1.3
Room temperature	24 h	99.41%	1.09

* SD—standard deviation; room temperature (25 ± 2 °C).

**Table 6:** Stability data for CYT in biological samples.

Type of Sample	Concentration (ng/mL)	Recovery (SD), n = 3			
		24 h in Room	48 h in 4 °C	30 Days in −20 °C	3 Freeze–Thaw Cycles
		Temperature			
Serum	1	88.95 (6.54)	97.12 (7.30)	94.01 (6.30)	86.99 (7.16)
	20	92.27 (4.97)	95.47 (6.18)	98.31 (4.33)	92.15 (9.00)
	180	87.90 (2.11)	96.73 (3.81)	97.05 (5.02)	86.88 (4.10)
Saliva	1	90.63 (6.96)	96.26 (7.25)	97.47 (6.76)	89.40 (8.11)
	10	93.17 (2.19)	93.20 (3.90)	99.03 (7.03)	90.17 (6.38)
	90	88.75 (3.11)	90.75 (5.75)	96.00 (4.46)	90.48 (6.07)

**Table 7:** The concentration of CYT in serum and saliva collected from an ENDS user, serum/saliva ratio, and predicted serum concentration of CYT in serum.

Day of Treatment	Time of Sample Collection (h)	Serum (ng/mL)	Saliva (ng/mL)	Saliva/Serum Ratio
1	0.5 ^1^	6.63	1.93	0.29
	1 ^1^	13.08	4.44	0.34
	2 ^1^	7.30	2.1	0.29
	4 ^1^	16.60	6.99	0.42
	6 ^1^	27.24	7.52	0.28
	8 ^1^	35.56	8.49	0.24
	10 ^1^	37.15	10.32	0.28
4	14 ^2^	12.44	3.17	0.25
			Mean:	0.30
			SD:	0.06
			%CV:	19.34

^1^ Time after the first drug dose of that day; ^2^ time after the last dose of the previous day.

## Data Availability

Data are available from the corresponding author upon reasonable request.

## Supplementary Materials

The following supporting information can be downloaded at https://www.mdpi.com/article/10.3390/ijms242015364/s1, references [37,38,39].
