# Supplementary material for: Development and Validation of LC-MS/MS Method for Determination of Cytisine in Human Serum and Saliva
Source: Int J Mol Sci. 2023 Oct 19;24(20):15364. doi: 10.3390/ijms242015364 (PMC10607646; doi:10.3390/ijms242015364)
Supplement: Supplementary file 1 [file ijms-24-15364-s001.zip › ijms-2660012-supplementary.pdf]

Development and validation of LC-MS/MS method for determination of cytisine in human serum and saliva

Karol Wróblewski[1,2,3], Małgorzata Szultka-Młyńska[4], Ryan J Courtney[5], Bogusław Buszewski[6], Piotr Tutka[2,5,7*]

1 Laboratory of Commercial and Non-Commercial Clinical Trials, University of Rzeszów, Kopisto 2a, 35-959 Rzeszow, Poland; kwroblewski@ur.edu.pl
2 Laboratory for Innovative Research in Pharmacology, University of Rzeszów, Kopisto 2a, 35-959 Rzeszow, Poland
3 Interdisciplinary Center for Preclinical and Clinical Research, University of Rzeszów, Werynia 2A, 36-100 Kolbuszowa, Poland
4 Department of Environmental Chemistry and Bioanalytics, Faculty of Chemistry, Nicolaus Copernicus University, Gagarina 7, 87-100 Torun, Poland; szultka.malgorzata@wp.eu
5 National Drug and Alcohol Research Centre, University of New South Wales, Sydney, NSW 2052, Australia; r.courtney@unsw.edu.au
6 Prof. Jan Czochralski Kuyavian-Pomeranian Science and Technology Center, Parkowa 1, 87-134 Przysiek, Poland; bbusz@chem.umk.pl
7 Department of Experimental and Clinical Pharmacology, University of Rzeszów, Kopisto 2a,35-959 Rzeszów, Poland
* Correspondence: ptutka@ur.edu.pl

Table S1. Characteristics of ENDS user treated with cytisine.

**Measurements**	**Visit**	
	**Visit 1: baseline data collection**	**Visit 2: 4 day of the treatment with cytisine**
Sex	male	
Race	white	
Age (years)	60	
Education	higher (university degree)	
Weigh	85 kg	
High	182 cm	
BMI	25.66 (overweight)	
RR [mmHg]	190/98	160/81
HR [1/min]	71	93
Respiratory rate [1/min]	18	17
Duration of e-cigarette use	5 years to date	
Smoking tobacco cigarettes	participant has not smoked for 5 years, and previously smoked cigarettes for 35 years since age 18	
Previous attempts to stop vaping	not reported	
Medical conditions	hypertension; generalized myasthenia gravis; condition after right hemisphere stroke (08.2016); left-sided limb paresis; discontinuous chroma; hypercholesterolemia; coronary stents	

<table>
<tr><td colspan="2">Current medication</td><td colspan="3">clopidogrel (75 mg per day), atorvastatin (20 mg per day), rosuvastatin (20 mg per day), ramipril (10 mg per day), bisoprolol (20 mg per day),vinpocetine (20 mg per day)</td></tr>
<tr><td colspan="2">Peripheral blood morphology</td><td colspan="2">no significant abnormalities found</td><td rowspan="6"></td></tr>
<tr><td rowspan="5">Biochemical blood test</td><td>Test</td><td>test result</td><td>reference range</td></tr>
<tr><td>ALT</td><td>21 U/L</td><td>< 33</td></tr>
<tr><td>AST</td><td>24 U/L</td><td>< 32</td></tr>
<tr><td>Creatinine</td><td>1.18 mg/dL</td><td>0.50 - 0.90</td></tr>
<tr><td>GFR</td><td>63 mL/min</td><td>> 60</td></tr>
<tr><td colspan="2">EKG</td><td colspan="2">sinus rhythm, normogram, moderate ST change, subendocardial ischaemia of the lateral wall</td><td>sinus rhythm, pathological rightogram, abnormal QRS-T axis angle, abnormal ST depression, anterolateral subendocardial ischaemia</td></tr>
<tr><td rowspan="3">Psychological questionnaires</td><td>Penn State Electronic Cigarette Dependence Index (PSCDI) [37]</td><td colspan="2">score: 8[1]</td><td>score: 10[2]</td></tr>
<tr><td>Depression Anxiety and Stress Scale (DASS 21) [38]</td><td colspan="2">score: 11[3]</td><td>score: 28[4]</td></tr>
<tr><td>The Alcohol Use Disorders Identification Test (AUDIT – C) [39]</td><td colspan="2">score: 3[5]</td><td>score: 3[5]</td></tr>
</table>

[1]Score 8 indicates low dependence; [2]score 10 indicates medium dependence; [3]score 11 indicates mild depression, moderate anxiety and normal stress; [4]score 28 indicates extremely severe depression and anxiety as well as severe stress; [5]score < 4 indicates lack of hazardous dringing or active alcohol use disorders.
